# The First Lumpy Skin Disease Outbreak in Thailand (2021): Epidemiological Features and Spatio-Temporal Analysis

**DOI:** 10.3389/fvets.2021.799065

**Published:** 2022-01-07

**Authors:** Orapun Arjkumpa, Minta Suwannaboon, Manoch Boonrod, Issara Punyawan, Supawadee Liangchaisiri, Patchariya Laobannue, Chayanun Lapchareonwong, Chaiwat Sansri, Noppasorn Kuatako, Pawares Panyasomboonying, Ponkrit Uttarak, Noppawan Buamithup, Chalutwan Sansamur, Veerasak Punyapornwithaya

**Affiliations:** ^1^Animal Health Section, The 4th Regional Livestock Office, Department of Livestock Development, Khon Kaen, Thailand; ^2^Animal Health Section, Roi Et Provincial Livestock Office, Department of Livestock Development, Bangkok, Thailand; ^3^Bureau of Disease Control and Veterinary Services, Department of Livestock Development, Bangkok, Thailand; ^4^Akkhararatchakumari Veterinary College, Walailak University, Nakhon Si Thammarat, Thailand; ^5^Veterinary Public Health and Food Safety Centre for Asia Pacific (VPHCAP), Faculty of Veterinary Medicine, Chiang Mai University, Chiang Mai, Thailand; ^6^Center of Excellence in Veterinary Public Health, Faculty of Veterinary Medicine, Chiang Mai University, Chiang Mai, Thailand

**Keywords:** lumpy skin disease, epidemiology, outbreak, spatio-temporal analysis, cattle farm, Thailand

## Abstract

The first outbreak of lumpy skin disease (LSD) in Thailand was reported in March 2021, but information on the epidemiological characteristics of the outbreak is very limited. The objectives of this study were to describe the epidemiological features of LSD outbreaks and to identify the outbreak spatio-temporal clusters. The LSD-affected farms located in Roi Et province were investigated by veterinary authorities under the outbreak response program. A designed questionnaire was used to obtain the data. Space-time permutation (STP) and Poisson space-time (Poisson ST) models were used to detect areas of high LSD incidence. The authorities identified 293 LSD outbreak farms located in four different districts during the period of March and the first week of April 2021. The overall morbidity and mortality of the affected cattle were 40.5 and 1.2%, respectively. The STP defined seven statistically significant clusters whereas only one cluster was identified by the Poisson ST model. Most of the clusters (*n* = 6) from the STP had a radius <7 km, and the number of LSD cases in those clusters varied in range of 3–51. On the other hand, the most likely cluster from the Poisson ST included LSD cases (*n* = 361) from 198 cattle farms with a radius of 17.07 km. This is the first report to provide an epidemiological overview and determine spatio-temporal clusters of the first LSD outbreak in cattle farms in Thailand. The findings from this study may serve as a baseline information for future epidemiological studies and support authorities to establish effective control programs for LSD in Thailand.

## Introduction

Lumpy skin disease (LSD) is an emerging viral disease that is known to affect cattle in several regions of Africa (1–3), Europe (4), and Asia (5, 6). It is caused by lumpy skin disease virus (LSDV), which belongs to the genus *Capripoxvirus* of the family *Poxviridae* (7). The LSDV can infect cattle, water buffaloes, and some wild ruminants (8). The disease is characterized by pyrexia, nasal discharge, swelling of the superficial lymph node, large skin nodules covering the entire body, poor milk production, and abortion (2, 8–11). The morbidity rate varies between 10 and 20%, whereas the mortality rate is generally low (1–5%) (5, 12, 13). It has also been revealed that the mortality and morbidity rates of LSD in cattle raised in naïve herds are typically greater than those raised in endemic settings (14). The main route of LSDV transmission is driven by arthropod vectors such as mosquitoes (14), ticks (15, 16), and stable flies (16–19).

LSD was first reported in Zambia in 1929 (2, 8) and thereafter spread to Sub-Saharan Africa, Middle Eastern countries, South-Eastern European countries, and Asian countries (9, 20). In Asia, the disease was first reported in Bangladesh in 2019 (21), followed by China (10), India (22), Nepal (23), Bhutan (9), Vietnam (24), Hong Kong (25), and Myanmar (9). Due to the potential risk of a rapid spread, the World Organization for Animal Health (OIE) has listed LSD as a notifiable disease (26).

Thailand reported the first confirmed LSD outbreak to the OIE official on April 4, 2021 (27). According to this report, the LSD outbreaks occurred in Roi Et province located in the northeastern region of Thailand during March and early April, 2021. Thereafter, the disease spread throughout the country, with many outbreaks recorded weekly, despite the fact that several control measures were implemented by livestock authorities, including the closure of cattle markets, restrictions on cattle trading and movements, insect control on farms, and cattle immunization (28). In response to the first LSD outbreak, livestock officers performed outbreak investigations in several districts. However, findings from the investigation have not yet been published. Additionally, the identification of outbreak clusters, which is critical for understanding the emergence and dissemination of LSD in the outbreak area, has not been documented.

The objectives of this study were (i) to describe the epidemiology of LSD in outbreak farms including clinical features of the LSD affected cattle, temporal trends of the LSD outbreaks and farm management practices related to the LSD prevention and controls, and (ii) to determine the LSD outbreak spatio-temporal clusters based on the outbreak investigation data regarding the first outbreak of LSD in Thailand.

## Materials and Methods

### Study Area and Case Definition

LSD outbreak investigations were officially undertaken in 293 farms located in the four districts of At Samat, Selaphum, Thung Khao Luang, and Panom Phrai, Roi Et province, north eastern Thailand between 1 March and 8 April, 2021. This province is well-known for having a large cattle population (Supplementary Figure 1). The investigations were based on the farmer notifications and active case detection by the authorities of Department of Livestock Development (DLD), Thailand. Accordingly, if farmers suspected LSD outbreaks and subsequently notified authorities, it was considered a passive approach. On the other hand, if authorities performed a survey of cattle farms in outbreak areas to identify possible LSD cases without receiving any notifications from farmers, this process was referred to as an active approach. In order to ensure that investigators had a thorough understanding of all of the questions being asked of the respondents, an outbreak investigation form was reviewed among the members of the investigation team before conducting outbreak investigations.

For the animal unit, a case was defined as individual cattle showing the LSD clinical signs of raised, circular, firm, nodules varying from 1 to 7 cm diameter without further laboratory confirmation (1, 8, 29). On the farm level, an outbreak farm was defined as a farm with at least one cattle diagnosed as an LSD case (1).

### LSD Outbreak Investigation

The investigations were performed by livestock officers and government veterinarians from the DLD. All LSD affected farms in the outbreak areas notified by active and passive approaches were included. Once a suspected case was notified, an outbreak investigation was undertaken on the same day or the next day. During the farm visit, the investigators interviewed farmers using the questionnaire designed explicitly for LSD outbreak investigation developed by the DLD authority. Farmers were interviewed and asked about the total number of cattle, the number of cattle showing clinical signs of LSD, general farm management practices, biosecurity measures implemented at their farms, and LSD affected cattle managements such as treatment, selling, and culling.

For clinical examination, a close inspection on cattle was performed by authorized veterinarians to explore the clinical signs of LSD. Inspections of cattle and barn areas were also carried out in order to assess whether any potential insect vectors, such as mosquitoes, flies, and ticks, were presented. There was no insect collection or quantification of insect abundance. During the outbreak investigations, geographical data including the latitude and longitude of each farm were also collected.

### Descriptive Analysis

Data management and descriptive analysis were conducted using R statistical software version 3.6.3 (30). Morbidity and mortality of LSD affected cattle, and percentage of LSD affected farm (herd attack rate) for each district were calculated. The morbidity rate was determined by dividing the number of cattle in LSD affected farms that showed clinical signs of LSD by the total number of cattle in those farms, whereas the mortality rate was defined as the number of cattle that showed clinical signs of LSD and then died from LSD divided by the total number of cattle in those farms. The herd LSD attack rate by the district was defined as the total LSD outbreak farms divided by the total farms in each district (31).

### Spatio-Temporal Analysis

Spatio-temporal analyses of the LSD outbreaks were carried out using a space-time permutation (STP) and Poisson space-time (Poisson ST) models (32) from SaTScan v.9.6 open-source software (33, 34). Both models utilize a dynamic cylindrical window, with a circular geographic base and with height corresponding to time to identify clusters (29, 35). These models test the null hypothesis of whether cases are randomly distributed over time and space (36).

The STP used information only from LSD-affected cattle (cases) from LSD outbreak farms (37, 38). Given that, the input variables for STP modeling included the number of LSD cases for each farm and the geographical coordinate of the farm. The STP was used to detect the presence of areas and time periods with the significant aggregation of LSD cases in LSD affected farms. Scanning for clusters included spatial and temporal dimensions ranging from 0 to 25% of the outbreak areas. Based on the incubation period of the disease (5, 39), the temporal unit was set as 7 days covering 5 weeks from 1 March to 14 April. The STP used case data within each candidate cylinder and calculated the ratio of the observed number of LSD cases to the expected number of LSD cases under the null hypothesis that observed cases are randomly distributed in space and time (40). The likelihood that a candidate cylinder represented a significant clustering of LSD cases was determined from the observed-to-expected ratio of LSD cases. The significance test to determine whether a cluster is formed by chance was carried out using the Monte Carlo simulation (number of replications = 999) (32, 40).

For the Poisson ST model, the number of LSD cattle cases and the number of total cattle in each farm, the coordinate of the farm, and the onset date of the outbreak were used as input variables with the assumption that cases in each farm have a Poisson distribution with a known population of cattle that are at risk for LSD (33). The spatial unit was made up of LSD-affected farms and the temporal unit was set at 7 days (5). The spatial size of the scanning window was defined to include 50% of the population at risk (41). The maximum time was also set as 50% of the total study period (29). A log-likelihood ratio (LLR) was calculated for each space-time window. Similar to the STP, a Monte Carlo simulation (number of replications = 999) was used to determine the statistical significance of detected clusters (33).

All maps were created by Quantum Geographic Information System (QGIS) which is an open-source software (42). Geographical data on administrative divisions were obtained from Chiang Mai University.

## Results

### LSD Outbreak Farms and LSD-Affected Cattle

**Figure 1:**
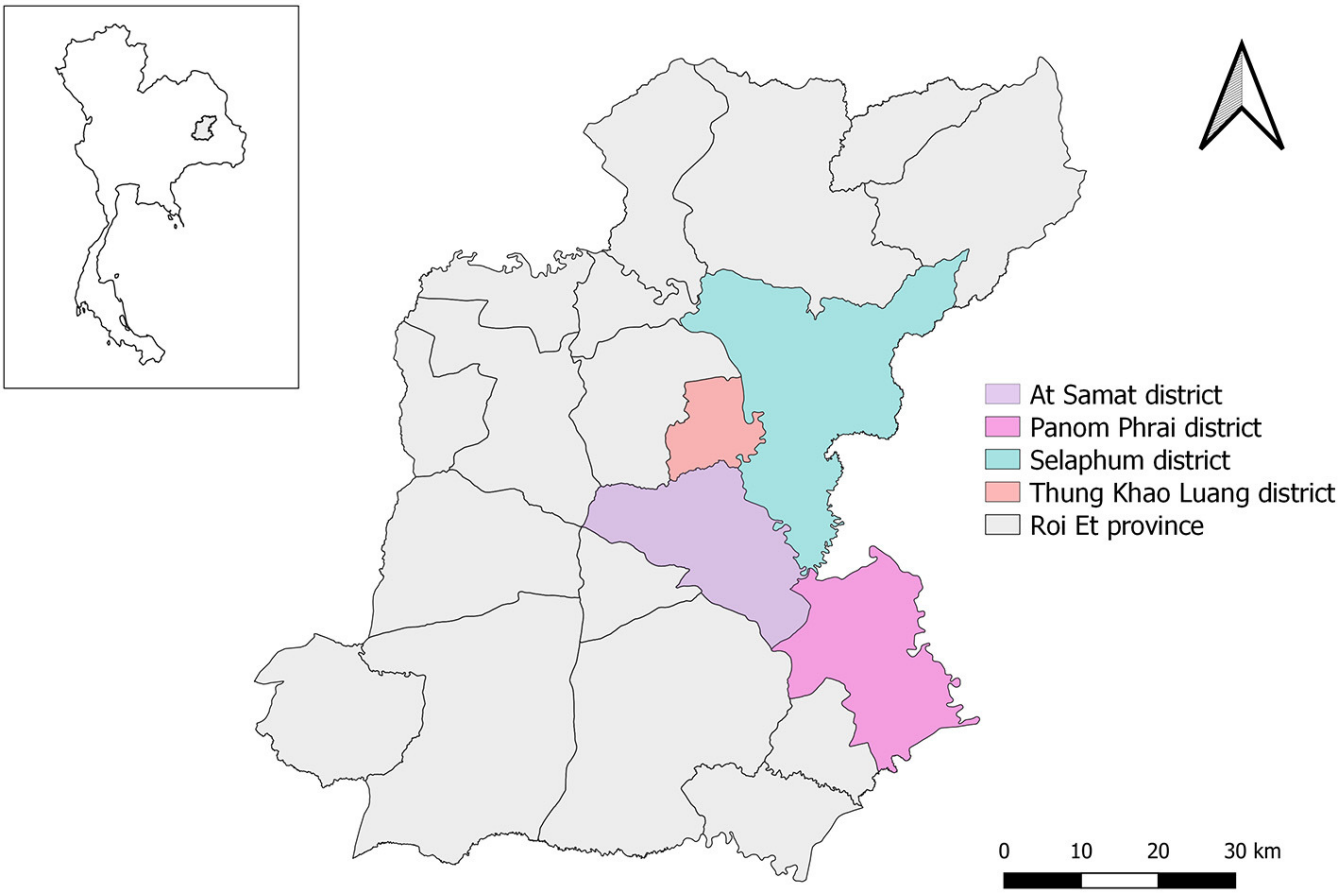
Study areas of LSD outbreak in four districts in Roi Et province, Thailand.

All the LSD affected farms were cattle farms located in Selaphum (*n* = 112), At Samat (*n* = 125), Thung Khao Luang (*n* = 52), and Panom Phrai (*n* = 4) (Figure 1) and were operated by smallholder farmers living in rural communities. The vast majority of farmers (97%, *n* = 283/293) own fewer than 10 head of cattle. The average total number of cattle was 4.3 head per farm. The mean number of cattle that showed clinical signs of LSD was 1.8 head per farm.

**Figure 2:**
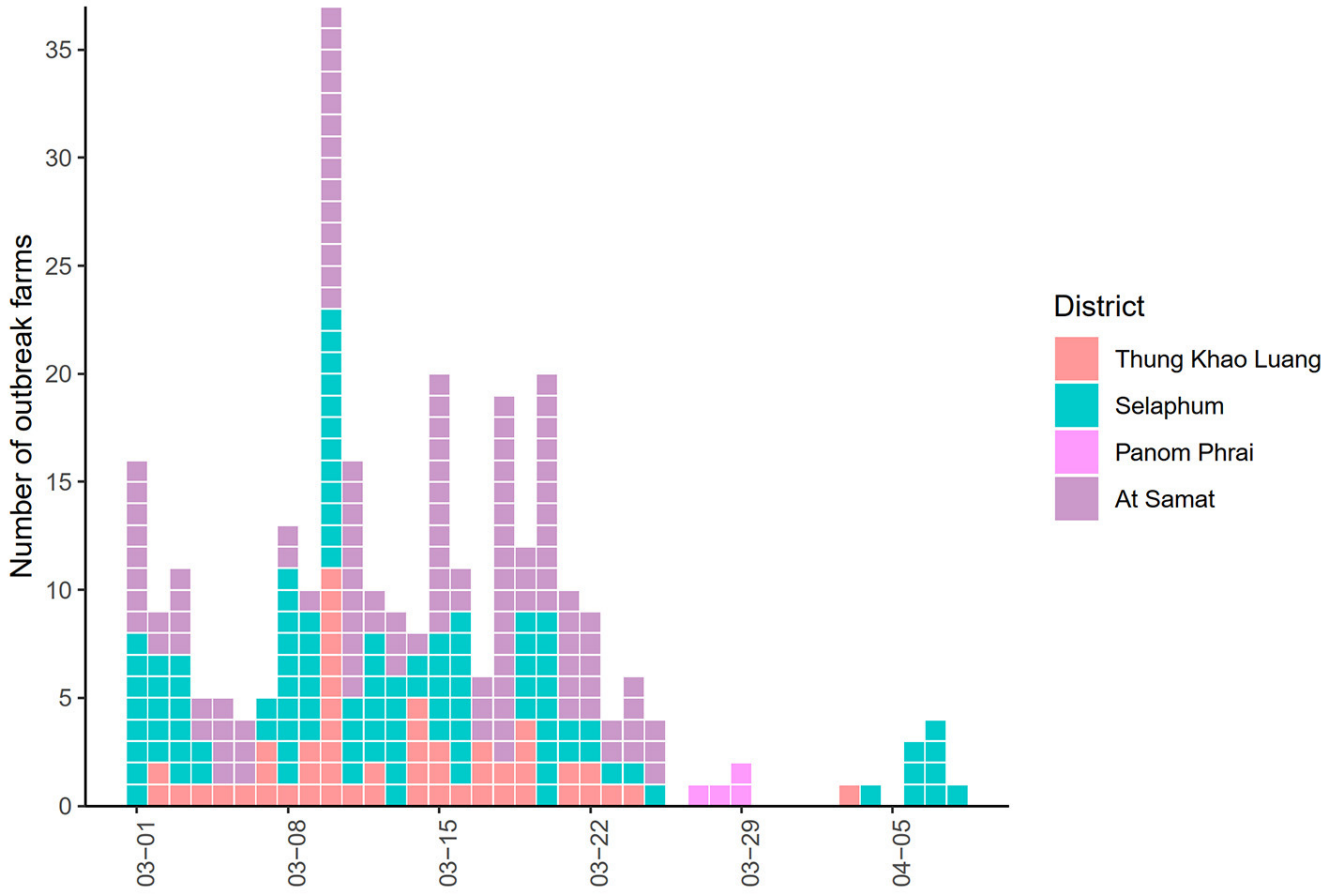
Epidemic curve of lumpy skin disease outbreaks in cattle farms in four districts of Roi Et province, Thailand.

The epidemic curve illustrated that the number of LSD outbreak farms varied over the study period (Figure 2). The highest number of outbreak farms (*n* = 37) were found on 3 March. After 25 March, the number of outbreak farms decreased to less than five each day. At the district level, the highest herd attack rate was found in Thung Khao Luang (5.2%; *n* = 52/988) followed by Selaphum (4.6%; *n* = 112/2,391), At Samat (3.4%, *n* = 125/3,637) and Panom Phrai districts (0.08%, *n* = 4/5,298), respectively.

**Figure 3:**
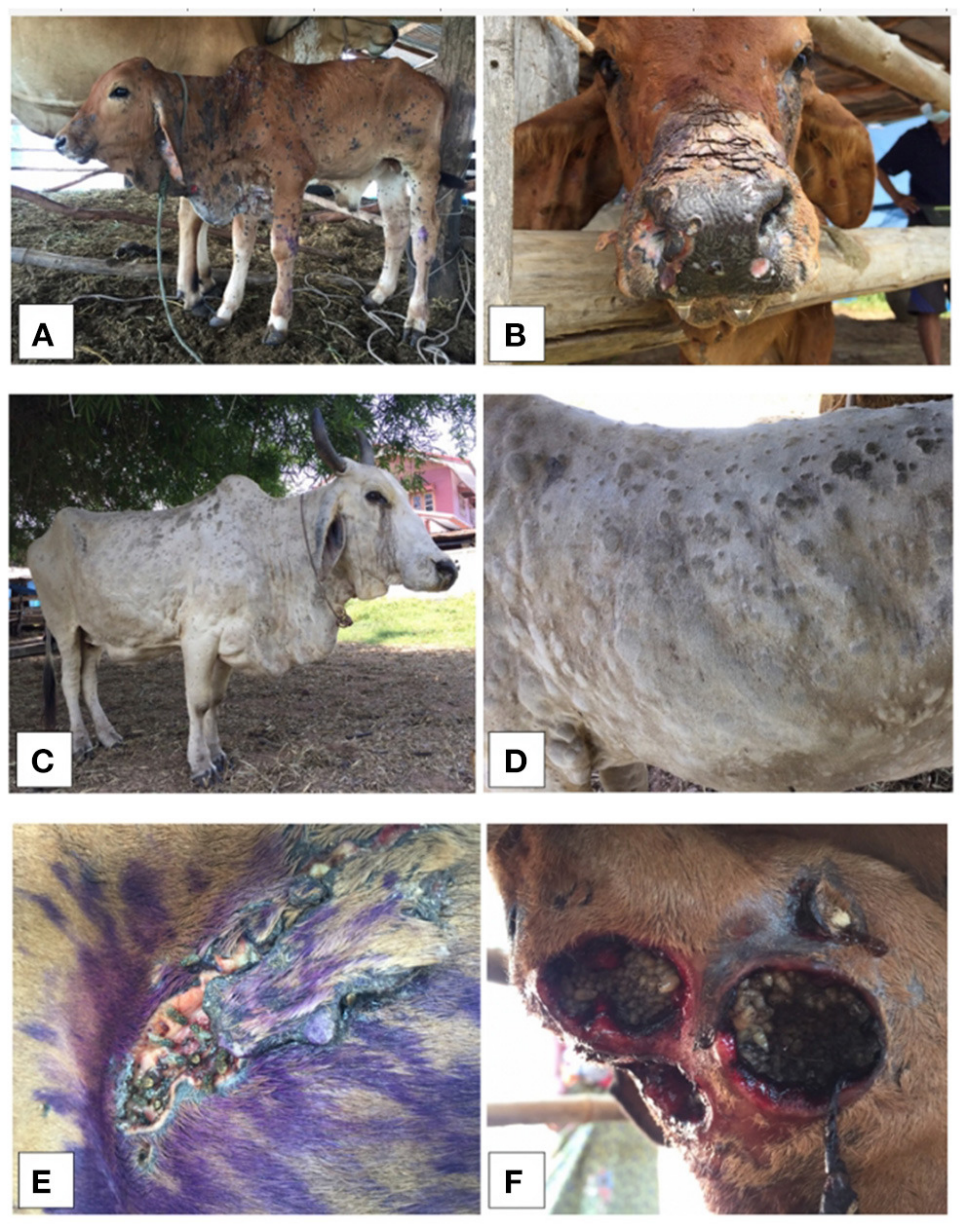
Clinical signs from lumpy skin disease (LSD) affected cattle (A-D) illustrating the generalized nodules in calf **(A,B)** and adult cattle **(C,D)**. The LSD complications of skin nodules with stable flies and maggots were also shown, respectively **(E,F)**.

Most LSD-affected cattle (95%) had nodules around the neck, leg, and flank areas (Figures 3A–D). LSD complications such as wounds with stable flies and maggots (Figures 3E,F) were observed in some cattle. The overall morbidity and mortality rates were 40.5% (*n* = 516/1,274) and 1.2% (*n* = 15/1,274), respectively. The overall morbidity and mortality rates by the districts are shown in Table 1.

**Table 1:** Morbidity and mortality rates of LSD affected cattle in LSD outbreak farms.

**District**	**Total number cattle in farms**	**Number of cattle show clinical sings**	**Number of death cattle**	**Morbidity (%)**	**Mortality (%)**
At Samat	589	228	6	38.7	1.0
Thung Khao Luang	215	78	3	36.3	1.4
Selaphum	458	200	5	43.7	1.1
Panom Phrai	12	10	1	83.3	8.3

All LSD cases were treated by either farm owners or veterinarians. None of the cattle showing clinical signs of LSD were sold during the outbreak period. All cattle that died due to LSD infection were buried under the oversight of the DLD authorities.

### Farm Biosecurity, Abundance of Insects and Insect Control

Forty percent of the cattle farms raised the cattle in the vicinity of the house while the rest allowed cattle to have free grazing or rotating them into different rice field areas. Only 4 out of 293 cattle farms (1.3%) had purchased new cattle 2 months prior to the outbreak. None of the cattle in the study areas were vaccinated against LSD.

Nearly 95% of the surveyed farms had at least one potential vector type (insect) responsible for LSDV transmission (Table 2). The most commonly found insects were mosquitoes and stable flies. According to the data, mosquitoes were found in 245 farms (84%), while stable flies were observed in 229 farms (78%). Nearly 25% of the farms had stable flies, mosquitoes, and tabanids. Also, about 17% of farms had all four types of insects. Most farmers repelled insects with the smoke generated from burning dried grass, while some used insect repellent light bulbs. A small proportion of farmers used insecticide spraying to control the insects. Table 3 shows insect control methods practiced by farmers.

**Table 2:** Primary pattern of insects found in lumpy skin disease outbreak farms.

**Stable flies**	**Mosquitoes**	**Tabanid**	**Hard tick**	***n***	**Percentage**
				72	24.6
				61	20.8
				51	17.4
				21	7.1
				20	6.8
				19	6.4
				16	5.4

*Green and gray colors indicate finding and not finding respectively*.

**Table 3:** Primary patterns of method using for insect control in lumpy skin disease affected farms.

**Smoke**	**Light bulbs**	**Spray**	***n***	**Percentage**
			151	51.5
			104	35.0
			17	5.8
			11	3.7
			10	3.4

*Green and gray colors indicate applicable and not applicable of a particular practice, respectively*.

### Spatio-Temporal Patterns

**Figure 4:**
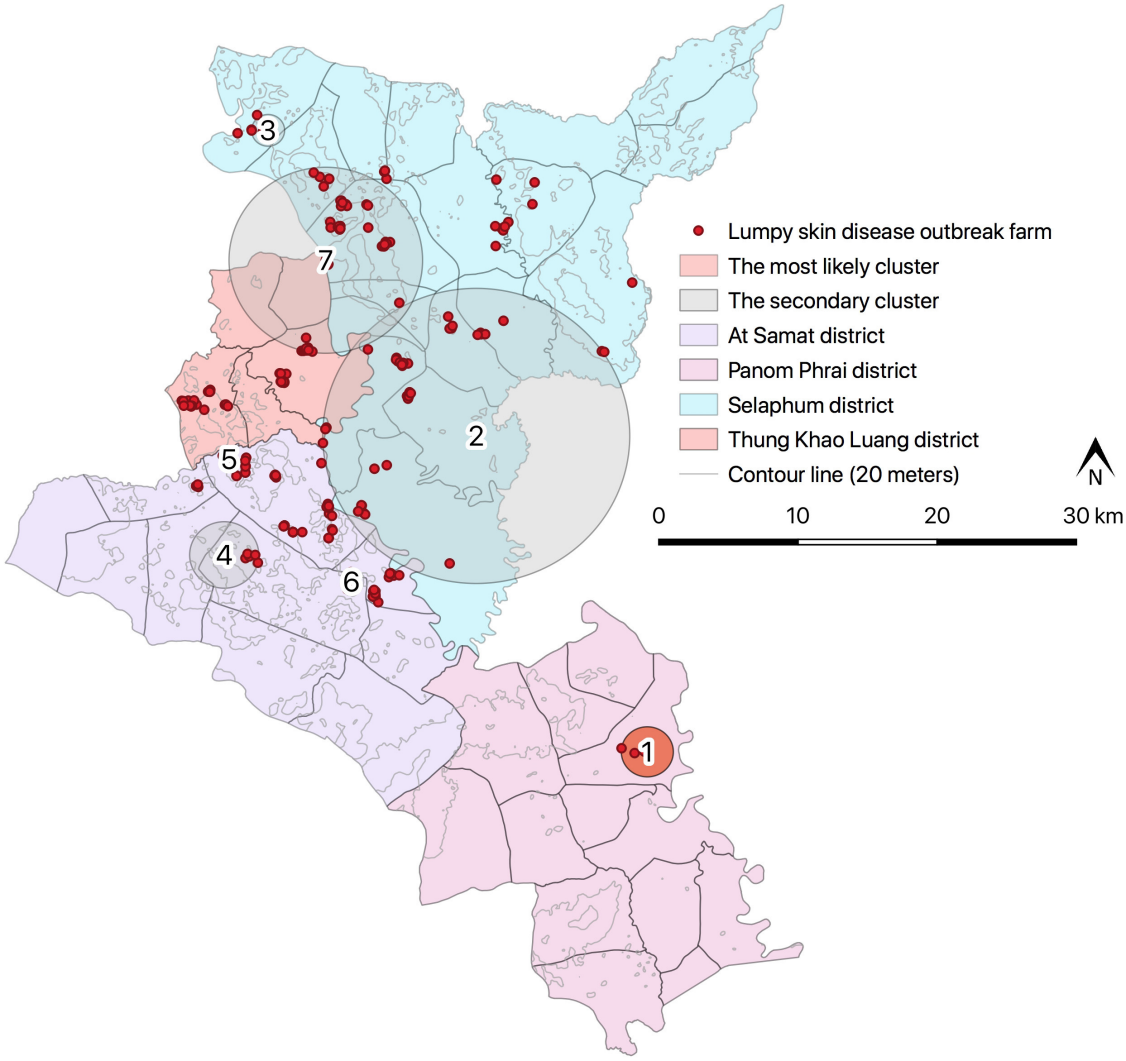
Spatio-temporal clusters of lumpy skin disease identified by space-time permutation model.

Results from STP model (Figure 4 and Table 4) revealed the most likely (*n* = 1) and secondary clusters (*n* = 6) with a total of 138 cases. The most likely cluster (Cluster 1) was found in Panom Phrai district between 27 March and 2 April, where LSD outbreak farms (*n* = 4) were located in an area with a radius of <2 km. Within Cluster 1, the number of cattle with LSD clinical sings (*n* = 10 cases) was greater than the expected number (approximately 1 case). The secondary clusters were found in cattle farms located in Selaphum (Cluster 2, 3, and 7), At Samat (Cluster 4, 5, and 6), and Thung Khao Luang districts (Cluster 2 and 7). The radii of these clusters ranged from <1 to 10.59 km, with the majority of clusters having a radius of <5 km.

**Table 4:** Spatio-temporal clusters by space-time permutation model on lumpy skin disease outbreaks in cattle farms in Roi Et, Thailand 2021.

**Cluster**	**Cluster time**	**Centroid (X, Y)/Radius (km)**	**O**	**E**	**O/E ratio**	**LLR**	***P*-value**
1^a^	27 Mar−2 Apr, 2021	(15.741942 N, 104.111556 E)/1.80 km	10	0.22	45.80	14.12	<0.001
2^b^	27 Feb−5 Mar, 2021	(15.945926 N, 104.001130 E)/10.59 km	45	18.24	2.47	14.71	<0.001
3^b^	3–9 Apr, 2021	(16.143228 N, 103.866746 E)/1.14 km	4	0.079	50.89	11.81	<0.001
4^b^	20–26 Mar, 2021	(15.869057 N, 103.838428 E)/2.39 km	12	2.27	5.28	10.35	<0.001
5^b^	27 Feb−5 Mar, 2021	(15.930841 N, 103.841493 E)/0.50 km	13	2.93	4.43	9.39	0.0013
6 ^b^	3–9 Apr, 2021	(15.851752 N, 103.920523 E)/less than 1 km	3	0.059	50.89	8.85	0.0026
7 ^b^	6–12 Mar, 2021	(16.059356 N, 103.903998 E)/6.67 km	51	28.10	1.81	8.11	0.0067

a *Most likely cluster;*

b *secondary cluster, O, observed case; E, expected case; O/E ratio, ratio of observed cases/expected cases; LLR, log likelihood ratio*.

There were two clusters (Cluster 2 and 7) including more than 40 LSD cases with radii ranging from 6.67 to 10.59 km. The cluster with the fewest cases was observed between 3 and 9 April, consisting of 3 cases from one LSD outbreak farm located in At Samart district (Cluster 6). Furthermore, Cluster 3 and 6 located in Selaphum and At Samat districts were observed at the same period (3–9 April). A similar finding was found in Clusters 2 and 5 located in Selaphum and At Samat districts respectively, where LSD cases were found from 27 February to 5 March.

**Figure 5:**
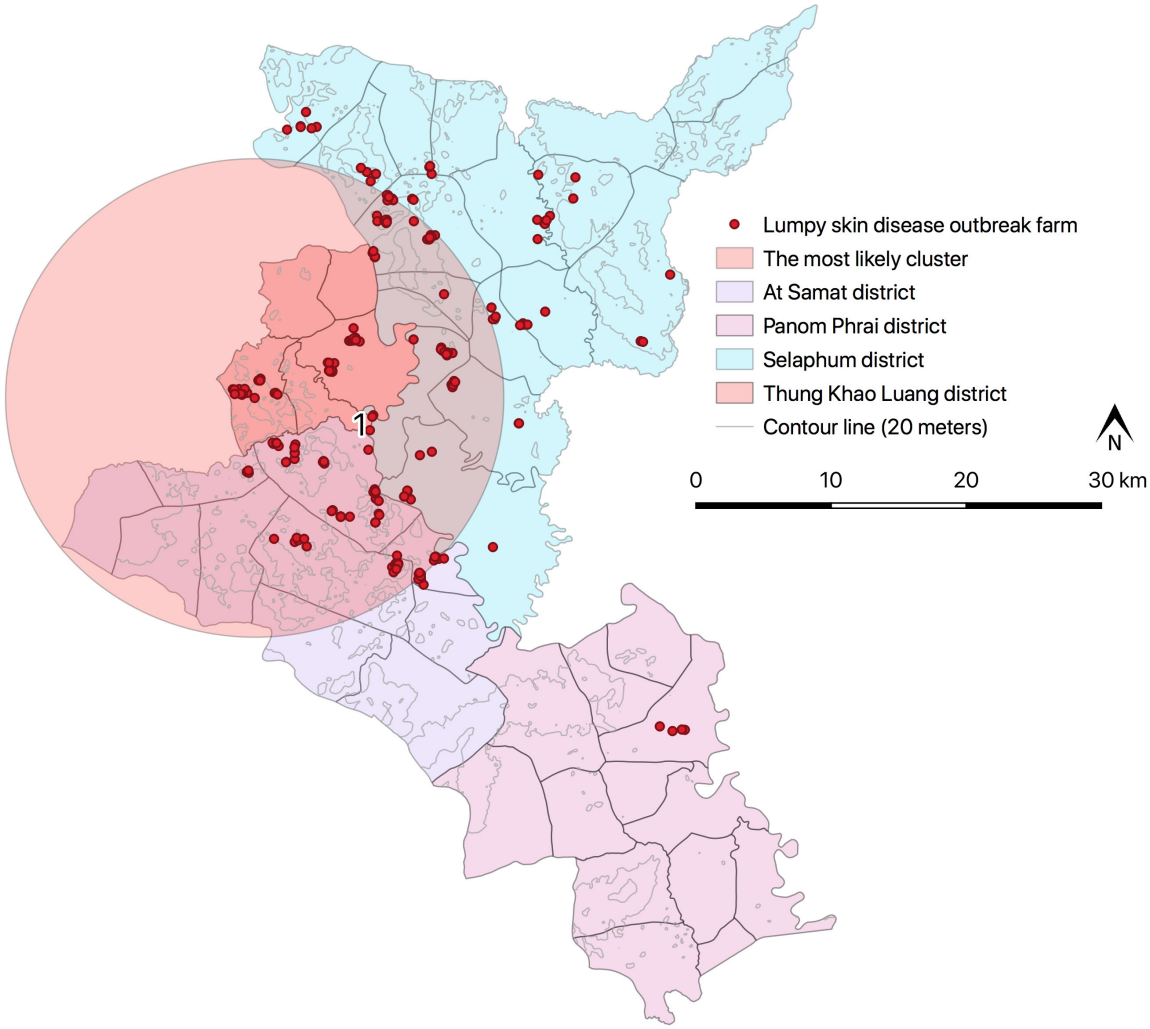
Spatio-temporal clusters of lumpy skin disease identified by Poisson space-time model.

The Poisson ST scan statistic model defined the most likely cluster of LSD outbreaks (Figure 5 and Table 5). This cluster had 198 LSD outbreak farms with 361 LSD cases during the period of 27 February−9 April. Due to a large radius (17.70 km), the most likely cluster included some areas of the three districts that shared boundaries. Moreover, compared to the largest cluster from the STP, the radius of the Poisson ST cluster was nearly twice as large.

**Table 5:** Spatio-temporal clusters by Poisson space-time scan statistic model on lumpy skin disease outbreaks in cattle farms in Roi Et, Thailand 2021.

**Cluster**	**Cluster time**	**Centroid (X, Y)/Radius (km)**	**O**	**E**	**O/E ratio**	**RR**	**LLR**	***P*-value**
1^a^	27 Feb−9 Apr, 2021	(15.962816 N, 103.825549 E)/17.70 km	361	53.29	6.77	28.27	552.09	<0.001

a *Most likely cluster; O, observed case; E, expected case; O/E ratio, ratio of observed cases/expected cases; RR, relative risk; LLR, log-likelihood ratio*.

## Discussion

LSD is currently considered as the most serious emerging disease affecting cattle in Thailand. Therefore, understanding the epidemiology of LSD to establish strategies to control and prevent the spread becomes critical for the authorities. The present study described the epidemiological features and identified spatio-temporal clusters of LSD in the outbreak areas.

The mortality and morbidity rates of LSD-affected cattle in this study are in accordance with those reported previously (2, 8–12, 29). Also, the clinical signs of LSD-affected cattle examined during the outbreak investigation were consistent with previous outbreak reports (43–45) such as the presentation of firm, slightly raised, circumscribed skin nodules that were 2–7 cm in diameter on their body areas such as the neck, legs, back, perineum or flank areas. Given that the most frequently observed pattern was the appearance of nodules on multiple areas of LSD-affected cattle, including the neck, leg, and flanks, it could be assumed that farmers and outbreak investigators could easily identify the clinical signs of LSD in affected cattle: thus, an identification of LSD-affected cattle in outbreak farms is straightforward. Furthermore, based on the epidemic curve, the occurrences of LSD outbreaks varied over the study period and were spatially dispersed. Thus, the spatial and temporal patterns of LSD outbreaks were further explored and determined.

The spatio-temporal analysis offers a visual output of the consequences of disease outbreaks, which can reflect the distribution and trend of disease spread in both space and time dimensions (29, 34). In this study, the spatio-temporal models were employed to detect clusters of LSD outbreaks according to the spatial and temporal features of the outbreaks. The results from STP revealed that the disease spread rapidly throughout the districts within a week. This finding could be linked to the fact that cattle farms were located in close proximity to one another resulting in the aggregation of the cases, which were then identified as disease clusters. Contrarily, the Poisson ST model defined only one cluster with a large radius and long duration, which may be explained by the fact that the number of cases and total cattle population in most farms in the study areas were similar. Indeed, the clusters revealed by the Poisson ST model support the DLD outbreak control program, implying that when an outbreak occurs on a farm, authorities should prioritize disease control in the surrounding areas falling within a 30 km radius. In practice, the clusters of LSD outbreaks defined in this study may help authorities concentrate their efforts on aggregated outbreak farms to gather additional information on the disease risks and spread. Management practices and other potential factors for the LSD epidemic can be compared between the farms within and outside the clusters. Also, livestock authorities can prioritize areas with a high number of aggregated cases for resource allocation and the implementation of stringent control measures.

Although the phylogenetic analysis from the previous outbreak alert report showed that the strain of LSDV collected from a cattle farm located in our study areas is closely related to isolates from Russia/2019, India/2019, and Kenya/2019 (27), there was no epidemiological data to demonstrate the source of the first LSD outbreak in Thailand. Most likely the LSD may have been introduced in Thailand by illegal movements of infected and carrier animals from the source country (28). The abundance of LSD insect vectors in cattle farms (46) and the inefficiency of insect control measures might further contribute to the spread of the disease (28, 47–50) in the outbreak areas. According to our results, stable flies and mosquitoes were commonly found in several LSD outbreak farms. Thus, if an outbreak occurred in one farm, there is a possibility that the LSDV was spread from one farm to other farms *via* such flying insects. We found that most farmers burned dried grass or rice straw with cattle manure during late evening and night to produce smoke that can repel flying insects. Some of the farmers used repellent light bulbs to deter the insects. However, these methods were not applicable during the daytime. Also, if the cattle are far from the source, smoke from burning dried grass and light bulbs may be ineffective. Thus, given the low frequency of live cattle movement among cattle farms and the abundance of insects in the study areas, we inferred that the disease was most likely spread by biting insects among cattle farms with insufficient insect control. Hence, authorities or veterinarians should encourage cattle farmers to improve their insect control measures to reduce disease transmission caused by insects.

Several studies have indicated vaccination as the most efficient way to limit the LSD spread (2, 51–54). Since there had been no previous LSD outbreaks in Thailand, our investigation data revealed that none of the cattle farmers had any experience with LSD vaccination of their cattle. Also, to our knowledge, no LSD vaccines were available in Thailand prior to the first outbreak. Yet, soon after the first emergence of LSD, the DLD procured live attenuated vaccines to control the LSD outbreaks in several cattle farming areas across the country (28). In addition to the vaccine campaign, we recommend that control measures such as restricting cattle movements and enhancing farm biosecurity should be strengthened (28).

Although this study provides comprehensive information on the epidemiological features of LSD in Thailand, some limitations need to be considered when interpreting the results. While we used the case definition similar to the previous LSD outbreak studies (1, 29), it is important to address that most LSD cases were diagnosed solely based on clinical findings. However, the likelihood of misdiagnosis may not be large because most cattle obviously showed clinical signs (e.g., more than 90% of cattle had nodules around the body) and the veterinary authorities who performed the diagnoses of LSD were trained before conducting the LSD outbreak investigation. Furthermore, there was a risk of under-detection of LSD cases because some LSD-affected cattle might not exhibit clinical signs during the investigation. Also, some farmers may be negligent in notifying the authorities of the outbreak and treating their cattle on their own, resulting in underestimating the outbreak occurrences. Moreover, we did not collect samples (e.g., blood or nodule tissue samples) from LSD-affected cattle; nevertheless, veterinarian authorities collected samples from some cattle showing clinical signs of LSD from some farms located in this outbreak area, and the results were documented in the outbreak alert report (27). In addition, it is important to note that some LSD outbreaks farms may have gone uninvestigated due to their remote locations. Furthermore, because some analyses were based on questionnaire data, there could have been some data collecting biases, such as recall and reporting biases, during data collection. Nonetheless, these biases may not significantly impact the analysis because the questionnaire survey was conducted shortly after outbreak notifications. Also, the questions were simple, and many items required only observation by investigators rather than responses from farmers.

LSD is an important new emerging disease in Thailand. Hence, a better understanding of this disease in the context of the country is necessary. The present study is the first report of our ongoing research project. Thus, follow-up studies on (i) occurrences of LSD in other outbreak locations or even across the country areas, (ii) risk factors of LSD outbreaks, (iii) economic impact of the disease on dairy farmers, and (iv) efficiency of LSD control program implemented are warranted.

## Conclusion

This was the first study to describe the epidemiology of the first LSD outbreak in Thailand based on the veterinary authority's survey. Spatio-temporal clusters of the disease were also identified providing advanced knowledge of spatial epidemiology of the disease, which is imperative for future disease prevention and control in the outbreak areas. Our findings are relevant in terms of understanding LSD emergence and spread in the first outbreak area, and these findings may be useful in developing effective surveillance and control measures.

## Data Availability Statement

The datasets generated for this study will not be made publicly available as the data has been provided by the authority of the Department of Livestock Development, Ministry of Agriculture and Cooperatives,Thailand. Requests to access these datasets or other materials (e.g., outbreak investigation form and questionnaire) should be directed to info@dld.go.th.

## Ethics Statement

Ethical review and approval was not required for the animal study because the authors used data from epidemic investigations conducted by authorized veterinarians as part of a disease outbreak response program regulated by the Department of Livestock Development, Thailand for this study.

## Author Contributions

OA, NB, and VP designed the study. OA, MS, SL, MB, IP, PL, CL, CS, NK, PP, and PU collected the data and carried out research works. OA, CS, and VP analyzed the data and prepared the initial draft of the manuscript. OA and VP provided oversight for the project. VP, NB, OA, and IP revised the manuscript. All authors read and approved the final manuscript.

## Funding

This work was partially funded by Chiang Mai University, Thailand. The funder had no role in the study design, data analysis, decision to publish, or manuscript preparation.

## Conflict of Interest

The authors declare that the research was conducted in the absence of any commercial or financial relationships that could be construed as a potential conflict of interest.

## Publisher's Note

All claims expressed in this article are solely those of the authors and do not necessarily represent those of their affiliated organizations, or those of the publisher, the editors and the reviewers. Any product that may be evaluated in this article, or claim that may be made by its manufacturer, is not guaranteed or endorsed by the publisher.
