# Supplementary figures and images for: The First Lumpy Skin Disease Outbreak in Thailand (2021): Epidemiological Features and Spatio-Temporal Analysis
Source: Front Vet Sci. 2022 Jan 7;8:799065. doi: 10.3389/fvets.2021.799065 (PMC8782428; doi:10.3389/fvets.2021.799065)

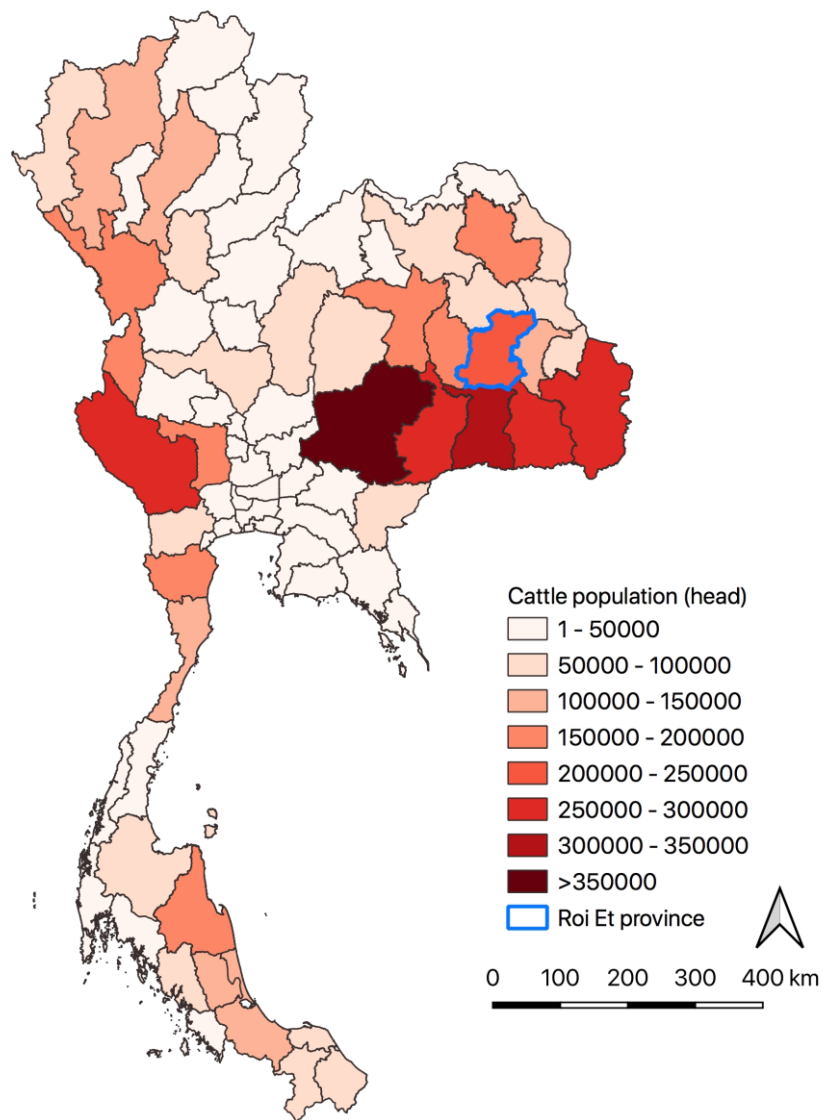

**Figure S1.** A map shows cattle population in Thailand

Supplement: Supplementary file 1 [file Image_1.PDF]
